# [La(η^*x*^-B_*x*_)La]^–^ (*x* = 7–9): a new class of inverse sandwich complexes†
†Electronic supplementary information (ESI) available. See DOI: 10.1039/c8sc05443f


**DOI:** 10.1039/c8sc05443f

**Published:** 2019-01-14

**Authors:** Teng-Teng Chen, Wan-Lu Li, Jun Li, Lai-Sheng Wang

**Affiliations:** a Department of Chemistry , Brown University , Providence , Rhode Island 02912 , USA . Email: Lai-Sheng_Wang@brown.edu; b Department of Chemistry , Key Laboratory of Organic Optoelectronics & Molecular Engineering of Ministry of Education , Tsinghua University , Beijing 100084 , China . Email: junli@tsinghua.edu.cn; c Department of Chemistry , Southern University of Science and Technology , Shenzhen , Guangdong 518055 , China

## Abstract

Despite the importance of bulk lanthanide borides, nanoclusters of lanthanide and boron have rarely been investigated. Here we show that lanthanide–boron binary clusters, La_2_B_*x*_^–^, can form a new class of inverse-sandwich complexes, [Ln(η^*x*^-B_*x*_)Ln]^–^ (*x* = 7–9). Joint experimental and theoretical studies reveal that the monocyclic B_*x*_ rings in the inverse sandwiches display similar bonding, consisting of three delocalized σ and three delocalized π bonds. Such monocyclic boron rings do not exist for bare boron clusters, but they are stabilized by the sandwiching lanthanide atoms. An electron counting rule is proposed to predict the sizes of the B_*x*_ ring that can form stable inverse sandwiches. A unique (d-p)δ bond is found to play important roles in the stability of all three inverse-sandwich complexes.

## Abstract 2

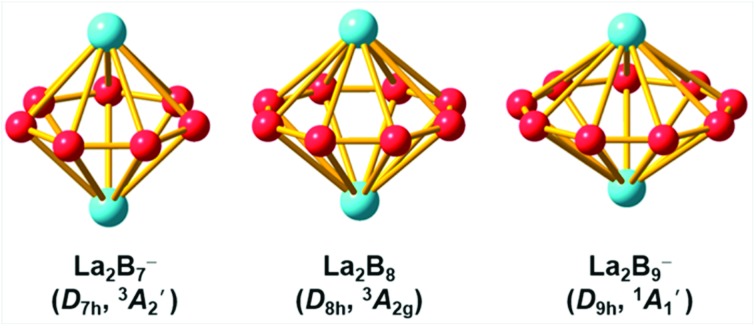
Photoelectron spectroscopy and computational chemistry reveal that lanthanide elements can form a class of novel inverse sandwich complexes consisting of aromatic B_7_, B_8_, and B_9_ monocyclic rings.

## 1. Introduction

Over the past decade, joint experimental and quantum chemistry investigations have shown that small boron clusters form planar structures, consisting of localized two-center-two-electron (2c-2e) σ bonds on the periphery of the clusters and delocalized σ and π bonding in the interior of the cluster plane due to the electron deficiency of the boron atom.^1^–^6^ The delocalized π bonding in the planar boron clusters has been shown to be analogous to aromatic hydrocarbons (arenes), giving rise to concepts of aromaticity and hydrocarbon analogues of boron clusters.^7^–^17^ However, while arenes can form sandwich compounds with transition metals due to strong d–π interactions,^18^–^20^ no similar sandwich complexes have been observed for the aromatic planar boron clusters. Because of the strong B–B bonds, such sandwich structures are energetically unfavourable, in comparison to the fusion of the two boron clusters. Instead, tubular-type or half-sandwich metal complexes of boron clusters have been observed.^21^–^26^ A particularly interesting class of transition metal doped boron clusters is the metal-centered borometallic molecular wheels, M©B_*x*_^–^ (*x* = 8–10), in which the monocyclic boron rings are stabilized by the central metal atoms,^27^–^33^ even though such boron rings are not stable by themselves.^7^,^8^ The largest ring size observed is B_10_ with M = Nb and Ta,^29^ while the smallest ring size is B_8_ for M = 3d transition metals.^27^,^30^ In fact, an even smaller B_7_ ring exists in the planar B_8_ cluster (*D*_7h_),^7^ which can be viewed as B©B_7_. The borometallic molecular wheels follow an electronic design principle, which requires double aromaticity in the σ and π frameworks.^27^,^31^,^33^ However, no such borometallic molecular wheels have been observed for lanthanide-doped boron clusters, for which only half-sandwich type structures have been observed.^34^,^35^ Recently, we have found that di-lanthanide B_8_ clusters (Ln_2_B_8_^–^) form unprecedented inverse-sandwich structures, which feature a doubly aromatic B_8_ monocyclic ring and strong (d-p)-π and -δ bonding between the B_8_ ring and the two Ln atoms.^36^ This recent study has shown that a variety of lanthanide elements can form the Ln(η^8^-B_8_)Ln inverse sandwiches (Ln = La, Pr, Tb). Two interesting questions arise: can similar inverse sandwiches be formed with other monocyclic boron ring sizes? What is the trend and nature of the bonding in such complexes?

Here we report the discovery of a series of La_2_B_*x*_^–^ clusters and an electron counting rule for predicting stable boron–metal inverse sandwiches. We have found that both La_2_B_7_^–^ and La_2_B_9_^–^ can form highly symmetric inverse-sandwich clusters, similar to La_2_B_8_^–^, while no other B_*x*_ ring size is likely to form such exotic structures. The global minimum structure of La_2_B_7_^–^ is found to have a triplet ground state with *D*_7h_ symmetry (^3^A_2_′), whereas that of La_2_B_9_^–^ is closed-shell with *D*_9h_ symmetry (^1^A_1_′). Chemical bonding analyses reveal that the La_2_B_*x*_^–^ clusters with *x* = 7–9 are highly stable with large HOMO–LUMO gaps and strong (d-p)-π and -δ interactions between the two La atoms and the boron rings. These highly symmetric and electronically stable clusters constitute a novel family of inverse-sandwich structures, [Ln(η^*x*^-B_*x*_)Ln]^–^ (*x* = 7–9), with tunable electronic and magnetic properties and may be viable for bulk syntheses with appropriate ligands. They also provide interesting motifs to design new lanthanide boride materials, such as one-dimensional nanowires.

## 2. Experimental method

The experiment was done using a magnetic-bottle type photoelectron spectroscopy (PES) apparatus coupled with a time-of-flight mass spectrometer and a laser vaporization cluster source, details of which has been published before.^5^,^37^ The laser vaporization target was prepared by first mixing La powder (Alfa Aesar, 99.9% purity) with B powder (Alfa Aesar, 96% ^11^B-enriched, 99.9% elemental purity) (5/2 La/B mass ratio) in a glove box and then pressing the mixture into a 12 mm diameter disc. The second harmonic of a Nd:YAG laser (532 nm) operated at a 10 Hz repetition rate was used for laser vaporization, synchronized with a high-pressure He carrier gas pulse seeded with 5% Ar. The ensuring nucleation led to the production of various La_*m*_B_*x*_^–^ clusters, which were entrained in the carrier gas and underwent a supersonic expansion. The size distribution and cooling of the clusters were controlled by the time delay between the vaporization laser pulse and the carrier gas pulse, as well as the resident time of the clusters inside the nozzle.^5^ The La_2_B_*x*_^–^ clusters of interest were mass-selected and decelerated before being photodetached by the 193 nm radiation from an ArF excimer laser. The photoelectron spectra were calibrated using the known spectrum of Bi^–^.^38^ The energy resolution of the apparatus was about 2.5%, that is, ∼25 meV for 1 eV electrons.

## 3. Theoretical methods

The global-minimum structures of La_2_B_7_^–^ and La_2_B_9_^–^ were searched using the Tsinghua Global Minimum (TGMin) program,^39^–^41^ which was based on a constraint Basin-Hopping algorithm.^42^ In total, more than 1700 minimal structures for La_2_B_7_^–^ and >2000 for La_2_B_9_^–^ with different spin multiplicities were evaluated using the ADF 2016 software.^43^ Scalar relativistic effects were taken into account in the calculations *via* zero-order regular approximation (ZORA).^44^ Density functional theory (DFT) with the PBE exchange–correlation functional^45^ and the TZP Slater-type basis sets^46^ were applied in the initial calculations. The frozen-core approximation was adopted for the [1s^2^] core of B and [1s^2^-4d^10^] core of La. All local minima were verified by calculations of the harmonic vibrational frequencies. Low-lying isomers within a PBE energy range of 100 kcal mol^–1^ were subsequently re-optimized using the hybrid PBE0 functional^47^ with the TZP basis set. To obtain even more accurate relative energies, we did further *ab initio* single-point CCSD(T) calculations for the three lowest-lying isomers using the Mopro 2012 package.^48^ The calculated T1 diagnostic factors of the CCSD calculations were 0.023 and 0.019 for the global minima of La_2_B_7_^–^ and La_2_B_9_^–^, respectively, suggesting that multi-reference features were not significant for these two systems. In the CCSD calculations, the cc-pVTZ valence triple-ζ basis set^49^ was used for B and the Stuttgart energy-consistent relativistic pseudopotential ECP28MWB(La) with the corresponding ECP28MWB_ANO basis set were used for La.^50^,^51^

The first ADE and first VDE were calculated at the PBE, PBE0, and CCSD(T) levels. In order to compare with the experimental PES data, we computed the higher VDEs approximately using the ΔSCF-TDDFT approach^52^ with the SAOP exchange–correlation functional.^53^ Chemical bonding was analysed using delocalized and localized MO approaches, as well as EDA-NOCV analyses.^54^,^55^ We also performed bonding analyses using the local coordinate system (LCS) of the B_7_ and B_9_ moieties, where the *z*-axis of each atom is pointed to the center of the ring and the *y*-axis is along the tangential direction. The chemical bonding was also analyzed using the adaptive natural density partitioning (AdNDP) approach^56^ at the PBE0/TZP level of theory. The charges of the atoms were calculated using various partition schemes, including Mulliken charge population,^57^ Hirshfeld,^58^,^59^ Voronoi^60^ and multipole-derived charge (MDC) approaches.^61^ The bond order indexes were calculated using the Mayer,^62^ Gopinathan–Jug (G–J),^63^ and Nalewajski–Mrozek^64^ schemes.

## 4. Experimental results

**Fig. 1:**
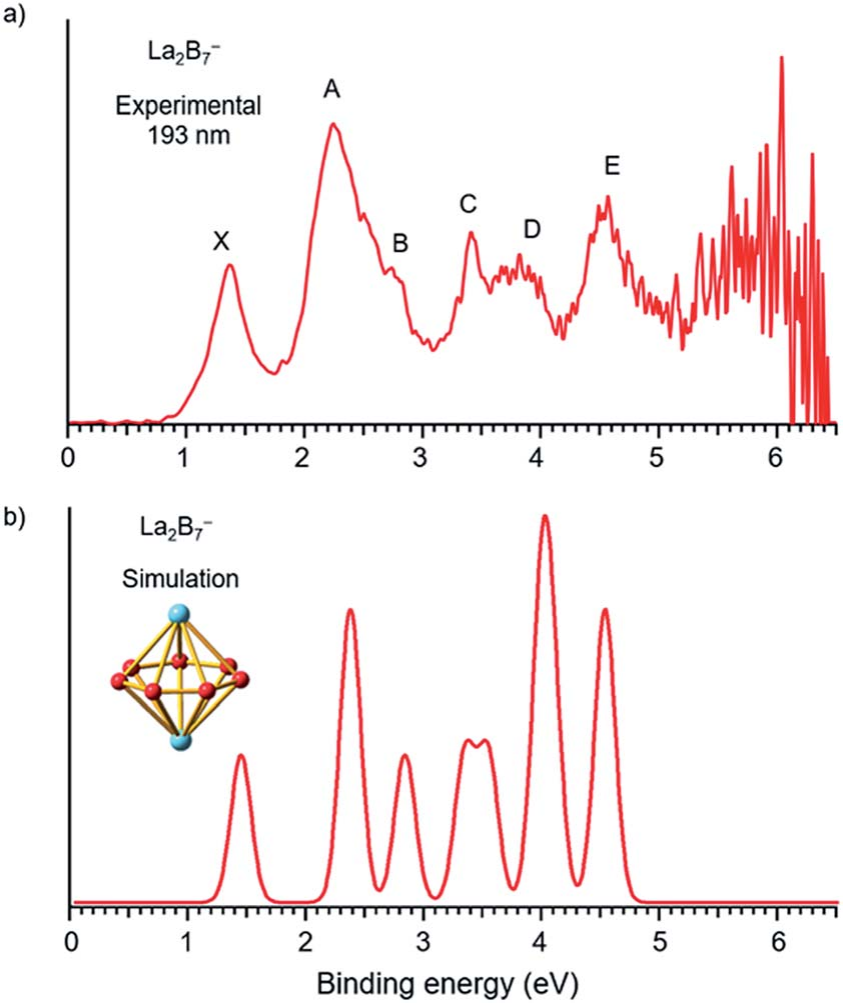
(a) Photoelectron spectra of La_2_B_7_^–^ at 193 nm (6.424 eV) and (b) the simulated spectrum for the *D*_7h_ global minimum of La_2_B_7_^–^, obtained by fitting the computed VDEs from Table 1 with Gaussians of 0.1 eV width.

**Fig. 2:**
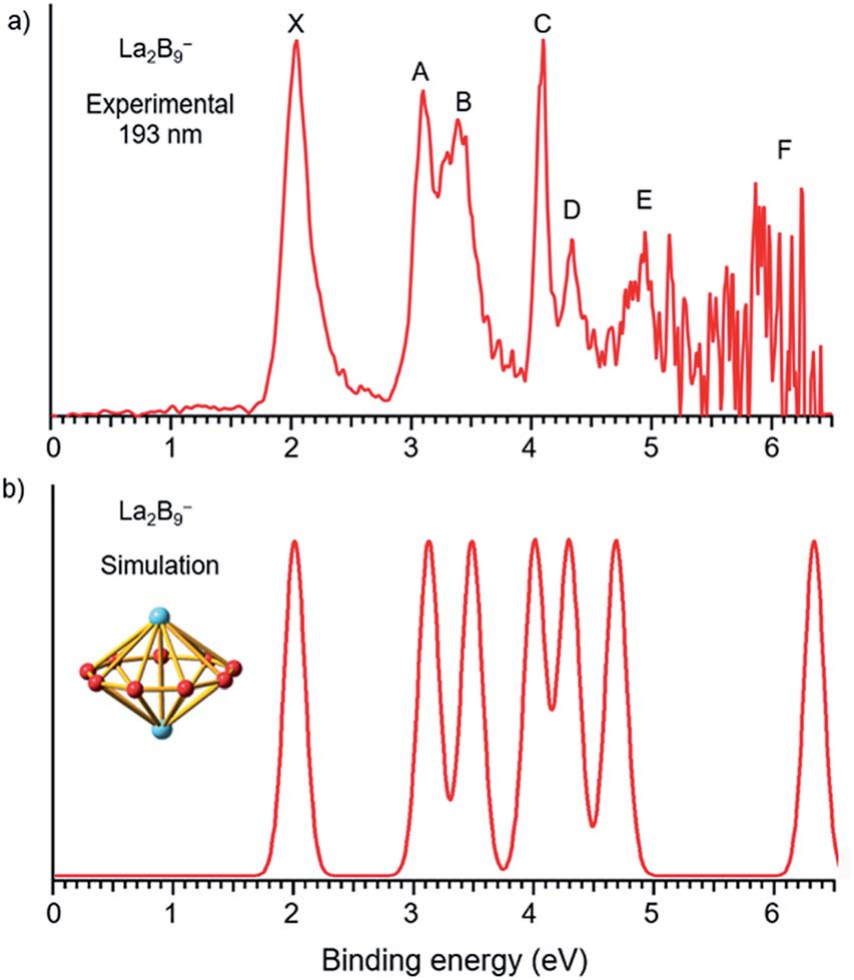
(a) Photoelectron spectra of La_2_B_9_^–^ at 193 nm (6.424 eV) and (b) the simulated spectrum for the *D*_9h_ global minimum of La_2_B_9_^–^, obtained by fitting the computed VDEs from Table 1 with Gaussians of 0.1 eV width.

The photoelectron spectra of La_2_B_7_^–^ and La_2_B_9_^–^ at 193 nm (6.424 eV) are shown in Fig. 1a and 2a, respectively. Major PES features are labeled with letters in Fig. 1a and 2a and the measured vertical detachment energies (VDEs) are given in Table 1, where they are compared with theoretical results to be presented below. The peak labeled X in each spectrum denotes the detachment transition from the ground electronic state of the respective anionic cluster to that of the corresponding neutral. The higher binding energy peaks (A, B, C…) represent detachment transitions from the ground state of the anion to excited electronic states of the neutral species.

**Table 1:** Experimental vertical detachment energies (VDE) from the photoelectron spectra of La_2_B_7_^–^ and La_2_B_9_^–^ in comparison with theoretical VDEs computed from the global minimum inverse sandwiches^*a*^

Feature	VDE^*b*^ (exp)	Final state and electronic configuration	VDE (theo.)
**La**_**2**_ **B**_**7**_^**–**^ **(*D***_**7h**_ **,** ^**3**^ **A**_**2**_ **′)**			
X	1.35(3)	^2^E_2_′′, {…1e_2_′^2^1e_3_′^4^3a_2_′′^2^4a_1_′^2^2e_1_′′^4^3e_1_′^4^**1e**_**2**_**′′**^**1**^}	1.40
A	2.22(3)	^2^E_1_′, {…1e_2_′^2^1e_3_′^4^3a_2_′′^2^4a_1_′^2^2e_1_′′^4^**3e**_**1**_**′**^**3**^1e_2_′′^2^}	2.31
		^4^E_1_′, {…1e_2_′^2^1e_3_′^4^3a_2_′′^2^4a_1_′^2^2e_1_′′^4^**3e**_**1**_**′**^**3**^1e_2_′′^2^}	2.33
B	2.81(4)	^4^E_1_′′, {…1e_2_′^2^1e_3_′^4^3a_2_′′^2^4a_1_′^2^**2e**_**1**_**′′**^**3**^3e_1_′^4^1e_2_′′^2^}	2.77
C	3.42(3)	^2^E_1_′′, {…1e_2_′^2^1e_3_′^4^3a_2_′′^2^4a_1_′^2^**2e**_**1**_**′′**^**3**^3e_1_′^4^1e_2_′′^2^}	3.28
D	∼3.8	^4^A_1_′′, {…1e_2_′^2^1e_3_′^4^**3a**_**2**_**′′**^**1**^4a_1_′^2^2e_1_′′^4^3e_1_′^4^1e_2_′′^2^}	3.47
		^2^A_2_′, {…1e_2_′^2^1e_3_′^4^3a_2_′′^2^**4a**_**1**_**′**^**1**^2e_1_′′^4^3e_1_′^4^1e_2_′′^2^}	3.91
		^4^A_2_′, {…1e_2_′^2^1e_3_′^4^3a_2_′′^2^**4a**_**1**_**′**^**1**^2e_1_′′^4^3e_1_′^4^1e_2_′′^2^}	3.93
		^2^A_1_′′, {…1e_2_′^2^1e_3_′^4^**3a**_**2**_**′′**^**1**^4a_1_′^2^2e_1_′′^4^3e_1_′^4^1e_2_′′^2^}	4.01
E	4.47(4)	^2^E_3_′, {…1e_2_′^2^**1e**_**3**_**′**^**3**^3a_2_′′^2^4a_1_′^2^2e_1_′′^4^3e_1_′^4^1e_2_′′^2^}	4.44
		^4^E_3_′, {…1e_2_′^2^**1e**_**3**_**′**^**3**^3a_2_′′^2^4a_1_′^2^2e_1_′′^4^3e_1_′^4^1e_2_′′^2^}	4.46
		^4^E_2_′, {…**1e**_**2**_**′**^**1**^1e_3_′^4^3a_2_′′^2^4a_1_′^2^2e_1_′′^4^3e_1_′^4^1e_2_′′^2^}	7.22
		^2^E_2_′, {…**1e**_**2**_**′**^**1**^1e_3_′^4^3a_2_′′^2^4a_1_′^2^2e_1_′′^4^3e_1_′^4^1e_2_′′^2^}	7.36

**La**_**2**_ **B**_**9**_^**–**^ **(*D***_**9h**_ **,** ^**1**^ **A**_**1**_ **′)**			
X	2.04(7)	^2^E_1_′′, {…1a_2_′^2^4e_1_′^4^3a_2_′′^2^5a_1_′^2^5e_1_′^4^2e_1_′′^4^**3e**_**1**_**′′**^**3**^}	1.98
A	3.09(9)	^2^E_1_′′, {…1a_2_′^2^4e_1_′^4^3a_2_′′^2^5a_1_′^2^5e_1_′^4^**2e**_**1**_**′′**^**3**^3e_1_′′^4^}	3.08
B	3.38(7)	^2^E_1_′, {…1a_2_′^2^4e_1_′^4^3a_2_′′^2^5a_1_′^2^**5e**_**1**_**′**^**3**^2e_1_′′^4^3e_1_′′^4^}	3.44
C	4.09(7)	^2^A_1_′, {…1a_2_′^2^4e_1_′^4^3a_2_′′^2^**5a**_**1**_**′**^**1**^5e_1_′^4^2e_1_′′^4^3e_1_′′^4^}	3.96
D	4.33(5)	^2^A_2_′, {…1a_2_′^2^4e_1_′^4^**3a**_**2**_**′′**^**1**^5a_1_′^2^5e_1_′^4^2e_1_′′^4^3e_1_′′^4^}	4.24
E	4.94(4)	^2^E_1_′, {…1a_2_′^2^**4e**_**1**_**′**^**3**^3a_2_′′^2^5a_1_′^2^5e_1_′^4^2e_1_′′^4^3e_1_′′^4^}	4.63
F	∼6.0	^2^A_2_′, {…**1a**_**2**_**′**^**1**^4e_1_′^4^3a_2_′′^2^5a_1_′^2^5e_1_′^4^2e_1_′′^4^3e_1_′′^4^}	6.43

^*a*^All energies are in eV.

^*b*^The numbers in the parentheses represent the uncertainty in the last digit.

### The photoelectron spectrum of La_2_B_7_^–^

The photoelectron spectrum of La_2_B_7_^–^ displayed in Fig. 1a exhibits six well-resolved bands labeled as X, A, B, C, D, E. Beyond 5 eV, the signal-to-noise ratio is quite poor and no PES bands can be definitively identified. The maximum of peak X at 1.35 eV yields the first VDE for La_2_B_7_^–^. Its first adiabatic detachment energy (ADE) is estimated to be 1.2 eV from the onset of band X, which also represents the electron affinity (EA) of the corresponding neutral La_2_B_7_. Band A at a VDE of 2.22 eV is intense and broad with a shoulder peak B discernible at 2.81 eV. Peak C at 3.42 eV is relatively sharp, followed closely by a broad band D at about 3.8 eV, which may consist of multiple detachment channels. Another relatively broad peak E is observed at 4.47 eV. These resolved PES features serve as electronic fingerprints, which can be compared with theoretical calculations (Fig. 1b, *vide infra*) to provide insights into the structures and bonding of La_2_B_7_^–^ and its corresponding neutral species.

### The photoelectron spectrum of La_2_B_9_^–^

Compared to the photoelectron spectrum of La_2_B_7_^–^, the spectrum of La_2_B_9_^–^ (Fig. 2a) is quite simple and all the PES features are much sharper, suggesting a stable and high symmetry structure. The strong ground state peak X yields the first VDE of 2.04 eV for La_2_B_9_^–^, and its leading edge gives an ADE of 1.94 eV, *i.e.*, the EA of the corresponding neutral La_2_B_9_. Following a large energy gap, peak A is observed at a VDE of 3.09 eV, closely followed by peak B at a VDE of 3.38 eV. Peak C at 4.09 eV is very sharp and intense, followed by a weaker peak D at 4.33 eV. Although the signal-to-noise ratios become poor in the high binding energy side, peak E at 4.94 eV is clearly resolved, whereas a peak F at around 6.0 eV can also be tentatively identified. The simplicity of the spectral pattern and the sharpness of the PES features for La_2_B_9_^–^ are surprising, suggesting not only a high symmetry structure, but also high structural stability both for the anion and for the corresponding neutral.

## 5. Theoretical results

### The global minimum of La_2_B_7_^–^

**Fig. 3:**
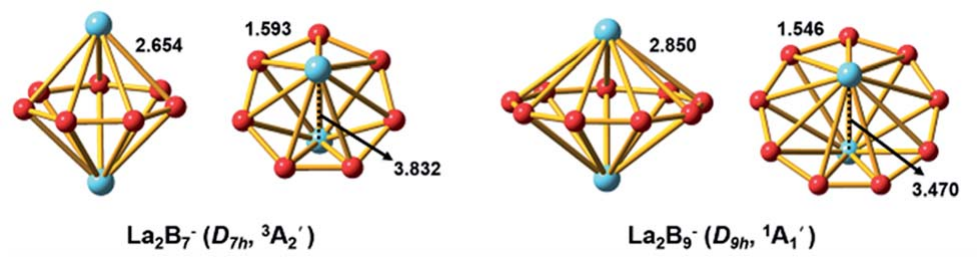
The optimized global-minimum inverse-sandwich structures of La_2_B_7_^–^ and La_2_B_9_^–^ at the PBE0/TZP level.

We performed an extensive global minimum search for La_2_B_7_^–^ using randomly-seeded structures with different spin multiplicities. In total, 1732 local minimum structures of La_2_B_7_^–^ were generated and the lowest 20 isomers are shown in Fig. S1.† Our calculations show that two inverse sandwich structures with different spin multiplicities are competing for the global minimum with all other isomers lying much higher in energy. The spin-triplet isomer I (^3^A_2_′) with perfect *D*_7h_ symmetry is found to be the global minimum at all three levels of theory, whereas the spin-singlet isomer II (^1^A_1_) with *C*_2v_ symmetry is found to be 3.03 kcal mol^–1^ higher in energy at the CCSD(T) level. In fact, these two isomers have very similar structures: the closed-shell isomer II (*C*_2v_, ^1^A_1_) has pseudo-*D*_7h_ symmetry, where the two La atoms are slightly off the C_7_ axis of the B_7_ ring to break symmetry. The spin-singlet isomer II with exact *D*_7h_ geometry is subject to Jahn–Teller distortions and is a saddle point (barrier ∼ 2.3 kcal mol^–1^) relative to the *C*_2v_ structure. The next isomer III lies much higher in energy, 25.98 kcal mol^–1^ at the CCSD(T) level, above the global minimum *D*_7h_ structure (Fig. S1†). Isomer XIII represents another type of inverse sandwich structure with a B-centered *D*_6h_-B_7_ motif and longer La–B bond lengths. However, this isomer is 56.04 kcal mol^–1^ higher in energy than the global minimum at the PBE0 level of theory. Two views of the global minimum of La_2_B_7_^–^ (*D*_7h_, ^3^A_2_′) with the relevant bond lengths are shown in Fig. 3.

### The global minimum of La_2_B_9_^–^

Because the La_2_B_9_^–^ cluster has more degrees of freedom than La_2_B_7_^–^, a much larger number of structures (>2000) were generated in the search for its global minimum using both the inverse sandwich as a seed and random seeds with different spin multiplicities. The lowest 20 isomers are given in Fig. S2.† Remarkably, a *D*_9h_ inverse sandwich structure with a monocyclic B_9_ ring and closed-shell electron configuration (^1^A_1_′) is found to be the global minimum for La_2_B_9_^–^. Two views of this structure with the relevant bond lengths are also given in Fig. 3. The closest isomer above the global minimum is found to have *C*_2v_ symmetry, being 10.43 kcal mol^–1^ higher in energy at the CCSD(T)/VTZ level. All other isomers are much higher in energy (Fig. S2†), suggesting the overwhelmingly high stability of the inverse sandwich structure for La_2_B_9_^–^.

As can be seen in Fig. 3, the B–B bond distances in the B_7_ and B_9_ rings of La_2_B_7_^–^ and La_2_B_9_^–^ are similar, with the former being slightly longer (1.593 Å). The La–B bond lengths (2.654 Å) in La_2_B_7_^–^ are much shorter than those in La_2_B_9_^–^ (2.850 Å). With the increase of the B_*x*_ ring size, the La···La and B–B distances decrease, and the La–B distances increase.

## 6. Comparison between experiment and theory

### The La_2_B_7_^–^ inverse sandwich

To validate the global minimum structure of La_2_B_7_^–^, we calculated its ADE and VDEs, as compared with the experimental data in Table 1 and Fig. 1b. The first ADE/VDE of the *D*_7h_ global minimum of La_2_B_7_^–^ were calculated at different levels of theory, as shown in Table 2. The computed first ADE/VDE of 1.35/1.40 eV at the CCSD(T) level are consistent with the experimental data of 1.2/1.35 eV. All the computed detachment channels and their comparison with the experimental data are given in Table 1. The simulated spectrum shown in Fig. 1b are obtained by fitting each detachment channel with a unit area Gaussian of 0.1 eV width.

**Table 2:** Experimental and theoretical first ADE and VDE, calculated from the PBE/TZP, PBE0/TZP, and CCSD(T)/VTZ methods for the global minimum inverse sandwich structures of La_2_B_7_^–^ and La_2_B_9_^–^

	ADE				VDE1			
	Exp	PBE	PBE0	CCSD(T)	Exp	PBE	PBE0	CCSD(T)
La_2_B_7_^–^	1.2	1.33	1.27	1.35	1.35	1.38	1.32	1.40
La_2_B_9_^–^	1.94	1.88	1.78	1.93	2.04	1.92	1.90	1.98

According to the MOs of La_2_B_7_^–^ shown in Fig. S3,† the ground state peak X is from electron detachment from the HOMO 1e_2_′′ orbital, which is half-filled with two unpaired electrons. Detachment from the HOMO-1 3e_1_′ orbital results in a low-spin and high-spin channel with similar VDEs, corresponding to the broad band A (Table 1). The calculated VDEs for the high-spin and low-spin channels for detachment from the HOMO-2 2e_1_′′ orbital are separated by ∼0.5 eV, consistent with peaks B and C, respectively (Table 1). Detachment from the HOMO-4 3a_2_′′ and HOMO-3 4a_1_′ orbitals give rise to four channels with relatively close VDEs, in agreement with the broad band D. Finally, the two detachment channels from the HOMO-5 1e_3_′ orbital have close VDEs of 4.44 and 4.46 eV, consistent with the broad band E at 4.47 eV. Overall, the simulated spectrum agrees well with the experimental data (Fig. 1), providing considerable credence to the *D*_7h_ inverse sandwich structure as the global minimum for La_2_B_7_^–^.

### The La_2_B_9_^–^ inverse sandwich

The first ADE/VDE for the La_2_B_9_^–^ inverse sandwich, calculated at different levels of theory, are also given in Table 2. The computed values of 1.93/1.98 eV at the CCSD(T) level are in excellent agreement with the experimental data of 1.94/2.04 eV. The computed VDEs for higher binding energy detachment channels are compared with the experimental values in Table 1. The simulated spectrum shown in Fig. 2b was obtained by fitting each computed detachment channel with a Gaussian of 0.1 eV width. Because the *D*_9h_ La_2_B_9_^–^ global minimum is closed shell, electron detachment from each MO (Fig. S4† and Table 1) yields a single detachment channel with a spin-doublet final state. Consequently, a very simple simulated spectral pattern is obtained, which is almost in quantitative agreement with the experimental spectrum, as revealed both in Fig. 2 and Table 1, unequivocally confirming the *D*_9h_ inverse sandwich global minimum for La_2_B_9_^–^. The sharpness of the La_2_B_9_^–^ PES features is quite surprising, which is evident of the extraordinary stability of the inverse sandwich structure for both the La_2_B_9_^–^ anion and the La_2_B_9_ neutral.

## 7. Discussions

### Structures and chemical bonding analyses

#### Localized MO analyses in the B_7_ and B_9_ rings

**Fig. 4:**
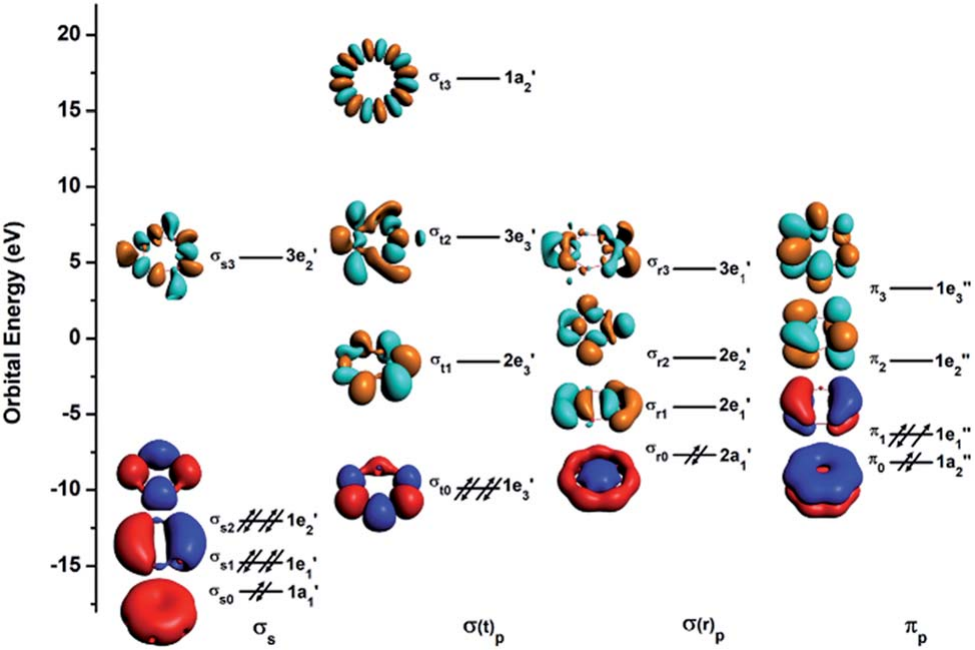
The localized coordinate system (LCS) analysis for the B_7_ ring at the PBE0/DZP level.

**Fig. 5:**
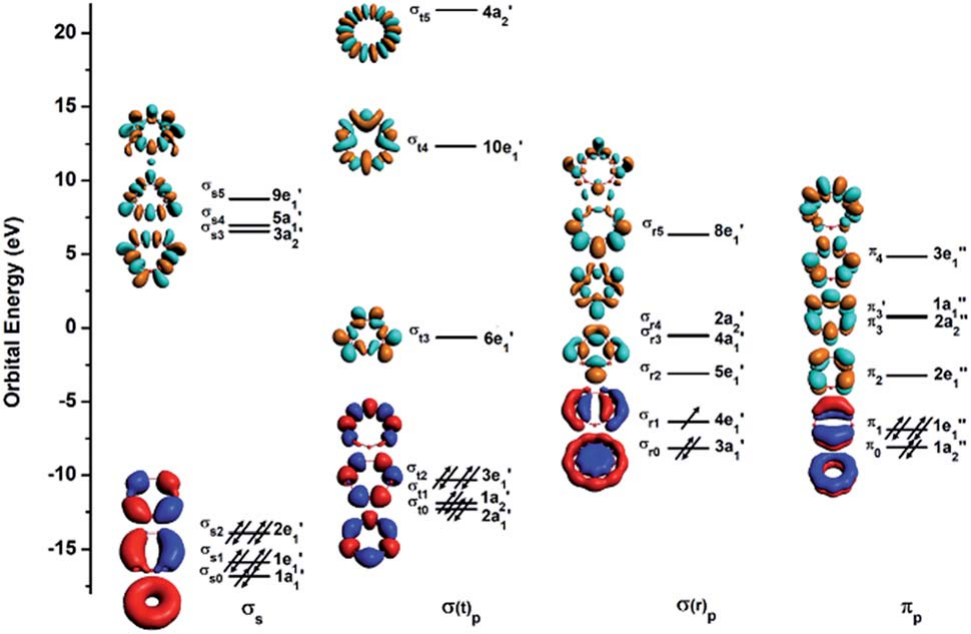
The LCS analysis for the B_9_ ring at the PBE0/DZP level.

To understand the exceptional stabilities of these inverse sandwich structures, we first investigate the local bonding in the B_7_ and B_9_ rings and see how they interact with the two La atoms. The LCS orbitals for the B_7_ and B_9_ rings are shown in Fig. 4 and 5, respectively. The B_7_ ring has 28 2s-2p valence orbitals and the B_9_ ring has 36 2s-2p valence orbitals. These orbitals can be divided into four categories using a tight-binding Hückel-type approach: σ_s_, σ(t)_p_, σ(r)_p_ and π_p_, where the (t) and (r) refer to tangential and radial bonding and the subscript indicates B 2s or 2p orbitals. There are similarities between the B_7_ and B_9_ rings. In both systems, the occupied σ_s_ and σ(t)_p_ orbitals are mainly responsible for the B–B bonds in the B_*x*_ rings and can be transformed to *x* 2c-2e B–B bonds (*vide infra*). The other two sets of orbitals, σ(r)_p_ and π_p_, are important in understanding the stability of the inverse sandwich structures; they are delocalized and mainly responsible for bonding with the two La atoms, as will be shown below. It should also be pointed out that the energy-level splitting for the σ(r)_p_ and π_p_ orbitals are smaller than that for the σ_s_ and σ(t)_p_ orbitals, mainly because the overlaps between the B atoms are smaller within the plane of the B_*x*_ rings, in comparison to the nearest neighbour B–B overlap around the ring periphery.

#### Chemical bonding in La_2_B_*x*_^–^ (*x* = 7–9)

**Fig. 6:**
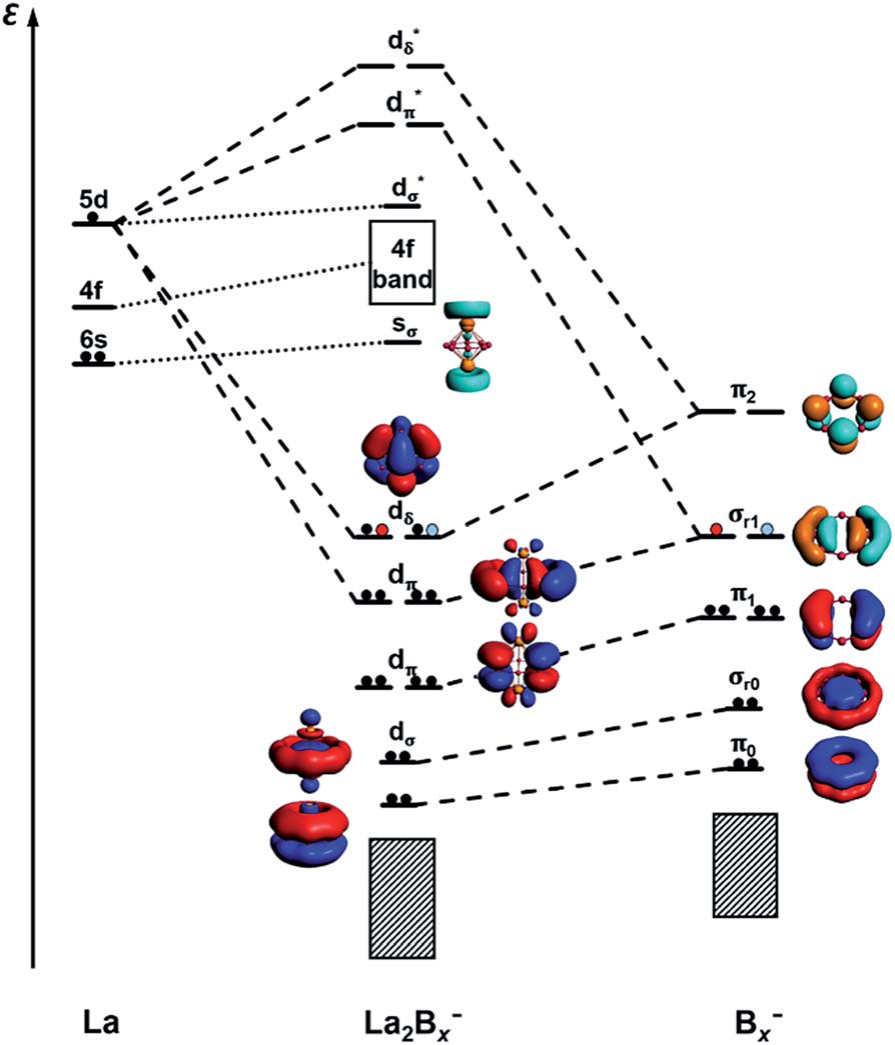
A schematic MO diagram for the La_2_B_*x*_^–^ (*x* = 7–9) inverse sandwiches, showing the major bonding interactions between the La 5d orbitals and the LCS orbitals of the B_*x*_^–^ ring. Red and blue dots indicate the successive additional electrons for La_2_B_8_^–^ and La_2_B_9_^–^, respectively.

It is clear now that we have a whole class of lanthanide inverse sandwiches with *x* = 7–9. Their bonding can be understood systematically on the basis of the interactions between the La···La atom pair and the LCS orbitals on the B_*x*_ ring moiety discussed above. Fig. 6 presents the schematic MO diagrams of La_2_B_*x*_^–^ (*x* = 7–9). The differences among La_2_B_7_^–^, La_2_B_8_^–^, and La_2_B_9_^–^ lie only in the occupation of the d_δ_ HOMO orbitals. The d_δ_ HOMO in La_2_B_7_^–^ is half-filled with two unpaired electrons, resulting in a triplet ground state, similar to the neutral La_2_B_8_ inverse sandwich. The additional electron in La_2_B_8_^–^ makes it a doublet ground state, leading to a Jahn–Teller distortion from *D*_8h_ in La_2_B_8_ to *D*_4h_ in La_2_B_8_^–^. In La_2_B_9_^–^, the d_δ_ HOMO is fully occupied, resulting in a closed-shell system and the exceptional stability for this large inverse sandwich. The stabilities of these inverse sandwich structures can be glimpsed from the large HOMO–LUMO gap revealed in the MO diagram of Fig. 6. The LUMO mainly originates from the La 6s orbitals, which only have weak interactions with the B_*x*_ rings. The 4f orbitals of the La atoms are also radially too contracted to contribute to chemical bonding with the B_*x*_ rings, so they form a nonbonding f-band just above the LUMO region. The 5d orbitals of the La atoms then play the most important role in bonding with the B_*x*_ ring in the inverse sandwich systems due to its large radial distribution and unique angular orientation. As can be seen in Fig. 6, the 5dδ orbitals of the La atoms are stabilized *via* bonding with the π_2_ orbital of the B_*x*_ ring, forming a novel (d-p)δ bonding-type for the HOMO, which has also been found previously in inverse sandwiches of uranium with arenes.^65^–^68^ The 5dπ orbitals overlap with the σ_r1_ orbital of the B_*x*_ ring – this (d-p)π bonding accounts for most of the interactions between La···La and the B_*x*_ ring. The 5dσ orbitals of the La atoms overlap with the σ_r0_ orbital of the B_*x*_ ring, and this (d-p)σ bonding-type also plays a tangible role in stabilizing the system.

We have further analyzed the detailed contributions of each bonding interaction using the method of energy decomposition analysis with the natural orbital for chemical valence (EDA-NOCV)^54^,^55^ for La_2_B_*x*_^–^ (*x* = 7–9), as summarized in Table S1.† The bonding between the La···La 5dπ orbitals and the B_*x*_ σ_r1_ orbitals, *i.e. E*_orb(1)_, is the most important component of the total orbital interactions (La_2_B_7_^–^: 74.8%; La_2_B_8_^–^: 64.8%; La_2_B_9_^–^: 46.4%), with electrons flowing from La_2_ to the B_*x*_^–^ fragment. The La_2_B_9_^–^ complex has the most contribution (42.4%) from the unique (d-p)δ bonding orbitals, *i.e. E*_orb(2)_, relative to La_2_B_7_^–^ and La_2_B_8_^–^, because of its full occupation. In these two major bonding interactions, *E*_orb(1)_ and *E*_orb(2)_, electrons are principally accumulated on the boron rings. The calculated charges and Mulliken spin distributions on the La and B atoms are consistent with the EDA-NOCV analyses, as shown in Table S2.† It is also worthy to mention that *E*_orb(3)_ and *E*_orb(4)_ depicted in Table S1† correspond to weak back donations from B_*x*_ π_1_ to La_2_ 5dπ and B_*x*_ σ_0_ to La_2_ 5dσ, respectively. These two interactions account for a small percentage of the total bonding, about 8.7% for La_2_B_7_^–^ and only about 5.5% for La_2_B_9_^–^.

#### AdNDP bonding analyses

**Fig. 7:**
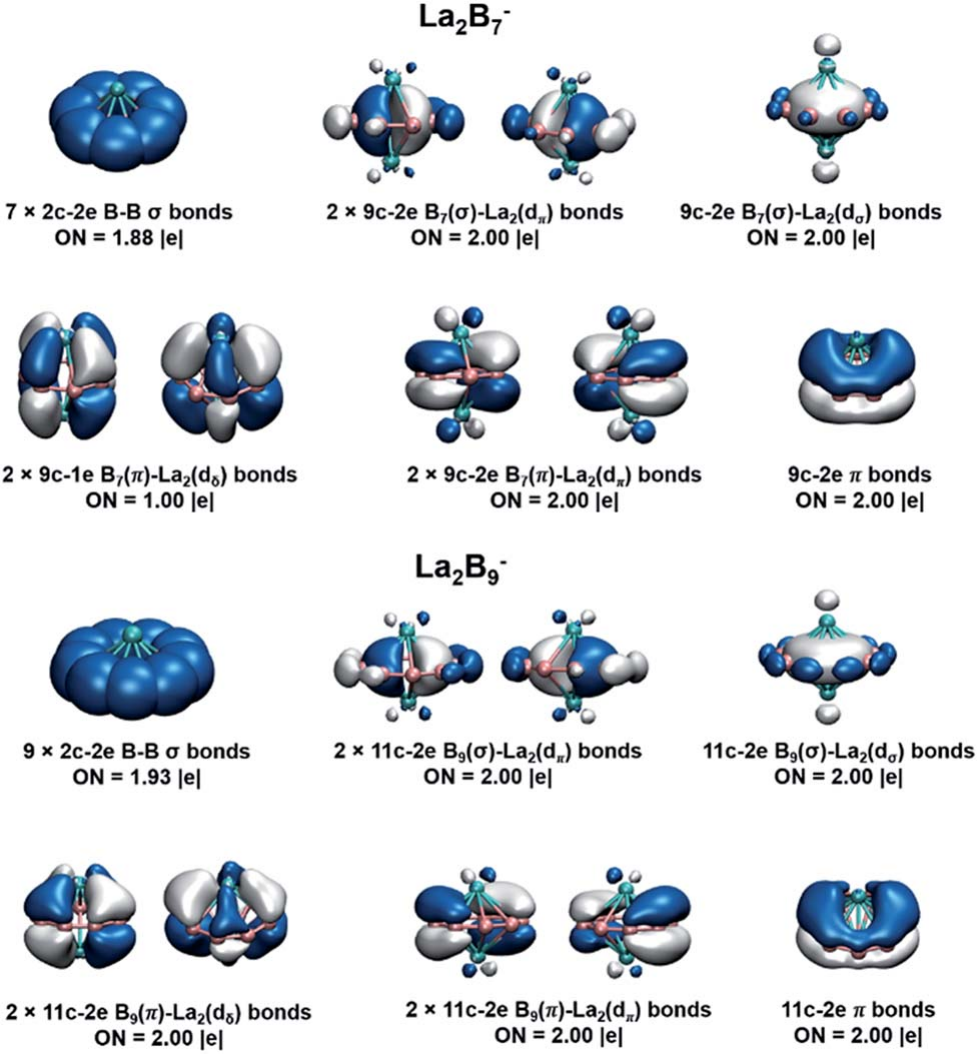
AdNDP analyses for the La_2_B_*x*_^–^ (*x* = 7, 9) inverse sandwiches at the PBE0/VTZ level. Occupation numbers (ON) are also given.

We further analyzed the bonding in La_2_B_7_^–^ and La_2_B_9_^–^ using the AdNDP method,^56^ as shown in Fig. 7. Four types of bonds are found for the inverse sandwiches: (1) *x* 2c-2e σ bonds on the periphery of the B_*x*_ ring; (2) three delocalized *n*c-2e σ bonds from the interactions between the La 5d_π_ and 5d_σ_ orbitals and the in-plane delocalized σ bonds within the B_*x*_ ring; (3) three delocalized *n*c-2e π bonds from the interactions between the La 5d_π_ and 5d_σ_ orbitals and the out-of-plane delocalized π bonds of the B_*x*_ ring; (4) two (d-p)δ bonds due to the interactions of the La dδ orbitals and the π orbitals of the B_*x*_ ring. The three delocalized *n*c-2e σ and π bonds give rise to double aromaticity for the inverse sandwiches, each satisfying the 4*n* + 2 Hückel rule. The only difference between the bonding in La_2_B_7_^–^ and La_2_B_9_^–^ lies at the (d-p)δ bonds. In La_2_B_7_^–^, the (d-p)δ orbitals (Fig. 6) are half-filled, resulting in two 9c-1e (d-p)δ bonds. This situation is exactly the same as in the neutral La_2_B_8_ inverse sandwich, which features two 10c-1e (d-p)δ bonds, whereas the La_2_B_8_^–^ anion contains one 10c-2e (d-p)δ bond and one 10c-1e (d-p)δ bond. In La_2_B_9_^–^, the (d-p)δ orbitals (Fig. 6) are completely filled, resulting in two 11c-2e (d-p)δ bonds. Thus, the stability of the inverse sandwiches is derived from the double aromaticity and the unique (d-p)δ bonds between the La atoms and the boron rings. The extraordinary stability of the La_2_B_9_^–^ inverse sandwich can be understood from the two full (d-p)δ bonds, whereas the La_2_B_7_^–^ and La_2_B_8_^–^ inverse sandwiches only have partial (d-p)δ bonds.

### 7.2. Stability of the inverse sandwich structures

To further quantify the stabilities of the La_2_B_*x*_^–^ (*x* = 7–9) inverse sandwiches, we examined the metal–metal interactions and the binding energies of the complexes, as shown in Table S3.† The binding energies were calculated as: La_2_B_*x*_^–^ → 2La + B_*x*_^–^. All three complexes show strong binding energies between the La atoms and the boron ring, increasing from 340.4 kcal mol^–1^ for *x* = 7 to 372.4 kcal mol^–1^ for *x* = 9. This trend is also consistent with the increasing (d-p)δ bond order, as discussed above. As the size of the B_*x*_ ring increases, the metal–boron distances become larger, while the distances between the two La atoms become smaller, indicating gradually weaker metal–boron interactions and stronger metal–metal interactions. As shown in Fig. 7, even though the La···La distance ranging from 3.83 to 3.47 Å is within the La–La single-bond length (3.60 Å based on the self-consistent covalent radius of Pyykkö),^69^ there is no clear La–La bond, which is reminiscent of the lack of metal–metal bonding in the Be_2_O_2_ rhombic structure.^70^,^71^ Instead, the bonding between the two La atoms and between the La atoms and the boron rings is completely by delocalized multi-center bonds. The stabilities of the inverse sandwiches depend mainly on the optimal overlaps between the La 5d orbitals and the 2p orbitals on the B_*x*_ ring. If the B_*x*_ ring is too large, no effective overlap is possible between the La 5d and the 2p orbitals on the B_*x*_ ring. Hence, La_2_B_9_^–^ is likely the largest inverse sandwich between lanthanide and boron. The B_10_ ring is probably too large to allow effective La–B interactions to form a stable La_2_B_10_^–^ inverse sandwich. Preliminary photoelectron data of La_2_B_10_^–^ showed a more complicated spectral pattern, incommensurate with a high symmetry structure. On the smaller side, it is more difficult to consider whether the B_6_ ring can form lanthanide inverse sandwich structures solely on the basis of the geometrical argument. Our preliminary experimental and theoretical data both suggest that it does not have the inverse sandwich global minimum structure.

Further insights obtained from the electronic structure and bonding of the La_2_B_*x*_^–^ (*x* = 7–9) inverse sandwiches also indicate that it would not be favorable for La_2_B_6_^–^ and La_2_B_10_^–^ to form inverse sandwiches. Fig. 7 shows that the stabilities of the inverse sandwiches derive from both the double aromaticity and the unique (d-p)δ bonds. Thus, in the [La(η^*x*^-B_*x*_)La] inverse sandwiches, we need 2(*x* + 6 + *y*) electrons, where 2*x* electrons for the bonding in the periphery of the B_*x*_ ring, 12 electrons for the double aromaticity and 2*y* electrons for the (d-p)δ bonds (*y* = 1, half-filled; *y* = 2, fully filled). According to the 2(*x* + 6 + *y*) rule, we would need 28 electrons for a closed-shell La_2_B_6_ inverse sandwich complex (*x* = 6; *y* = 2), but La_2_B_6_ only has 24 valence electrons, which means that there would be no more electrons for the (d-p)δ bonds. For the La_2_B_6_^–^ anion, there would be only one electron for the (d-p)δ bonds, which explains the instability of a La_2_B_6_^–^ inverse sandwich structure. It is interesting to note that the Ta_2_B_6_ cluster with 28 valence electrons has been found previously to form a highly stable *D*_6h_ Ta(η^6^-B_6_)Ta inverse sandwich.^72^ A similar electronic consideration suggests that a La_2_B_10_^–^ inverse sandwich would have one extra electron, which would occupy the high energy 6s-based LUMO (Fig. 6), making it energetically unfavorable. Hence, we conclude that La_2_B_*x*_^–^ (*x* = 7–9) would be the only likely inverse sandwich complexes in term of the size of the B_*x*_ ring. On the other hand, our previous work showed that the Ln_2_B_8_^–^ species can form inverse sandwiches for a range of lanthanide elements for Ln = La, Pr, and Tb.^36^ Therefore, we expect that most lanthanides should also be able to form the inverse sandwiches in the same size range, [Ln(η^*x*^-B_*x*_)Ln]^–^ (*x* = 7–9). Given the diverse magnetic properties of the lanthanides, the Ln_2_B_*x*_^–^ (*x* = 7–9) clusters constitute a novel class of inverse sandwich complexes with tunable chemical and physical properties. They not only provide new motifs for bulk borides, but it is also conceivable that some of these inverse sandwiches may be able to be synthesized in solution with appropriate ligand coordination, similar to the actinide arene inverse sandwiches.^65^–^68^

## 8. Conclusions

We report the discovery of a new class of di-lanthanide boron inverse sandwich complexes: [Ln(η^*x*^-B_*x*_)Ln]^–^ (*x* = 7–9). Photoelectron spectroscopy of La_2_B_7_^–^ and La_2_B_9_^–^ revealed relatively simple spectral patterns, suggesting highly symmetric structures for the two cluster anions. Global minimum searches found that these two clusters form highly stable inverse sandwich structures. Simulated photoelectron spectra are in excellent agreement with the experimental data, confirming the high symmetry and stability of the inverse sandwich structures. Strong delocalized chemical bonding is found in the inverse sandwiches, involving the La 5d orbitals and the 2p-based orbitals of the boron ring moiety *via* double (σ and π) aromaticity and strong (d-p)δ interactions. Geometrical, chemical bonding, and electronic structure analyses suggest that the size range of the di-lanthanide boron inverse sandwiches is likely to be limited to *x* = 7–9 for [Ln(η^*x*^-B_*x*_)Ln]^–^, which form a novel class of lanthanide–boron complexes with potentially tunable chemical and physical properties. These inverse sandwich structures provide opportunities to design new types of lanthanide–boride materials, such as 1D chain-like nanowires, and they may also be synthesized as new inorganic compounds with appropriate ligand protections.

## Conflicts of interest

The authors declare no conflict of interest.

## Supplementary Material

Supplementary information
