# Supplementary material for: [La(ηx-Bx)La]– (x = 7–9): a new class of inverse sandwich complexes
Source: Chem Sci. 2019 Jan 14;10(8):2534–42. doi: 10.1039/c8sc05443f (PMC6385817; doi:10.1039/c8sc05443f)
Supplement: Supplementary file 1 [file SC-010-C8SC05443F-s001.pdf]

Electronic Supplementary Material (ESI) for Chemical Science.
This journal is © The Royal Society of Chemistry 2019

Supporting Information

$[La(\eta^{x}\text{-}B_{x})La]^{-}$ (x = 7-9): A New Class of Inverse Sandwich Complexes

Teng-Teng Chen, ‡[a] Wan-Lu Li, ‡[b] Jun Li,*[bc] and Lai-Sheng Wang *[a]

[a] Department of Chemistry, Brown University, Providence, Rhode Island 02912, USA.

[b] Department of Chemistry and Key Laboratory of Organic Optoelectronics & Molecular Engineering of Ministry of Education, Tsinghua University, Beijing 100084, China.

[c] Department of Chemistry, Southern University of Science and Technology, Shenzhen, Guangdong 518055, China.

‡These authors contribute to this article equally.

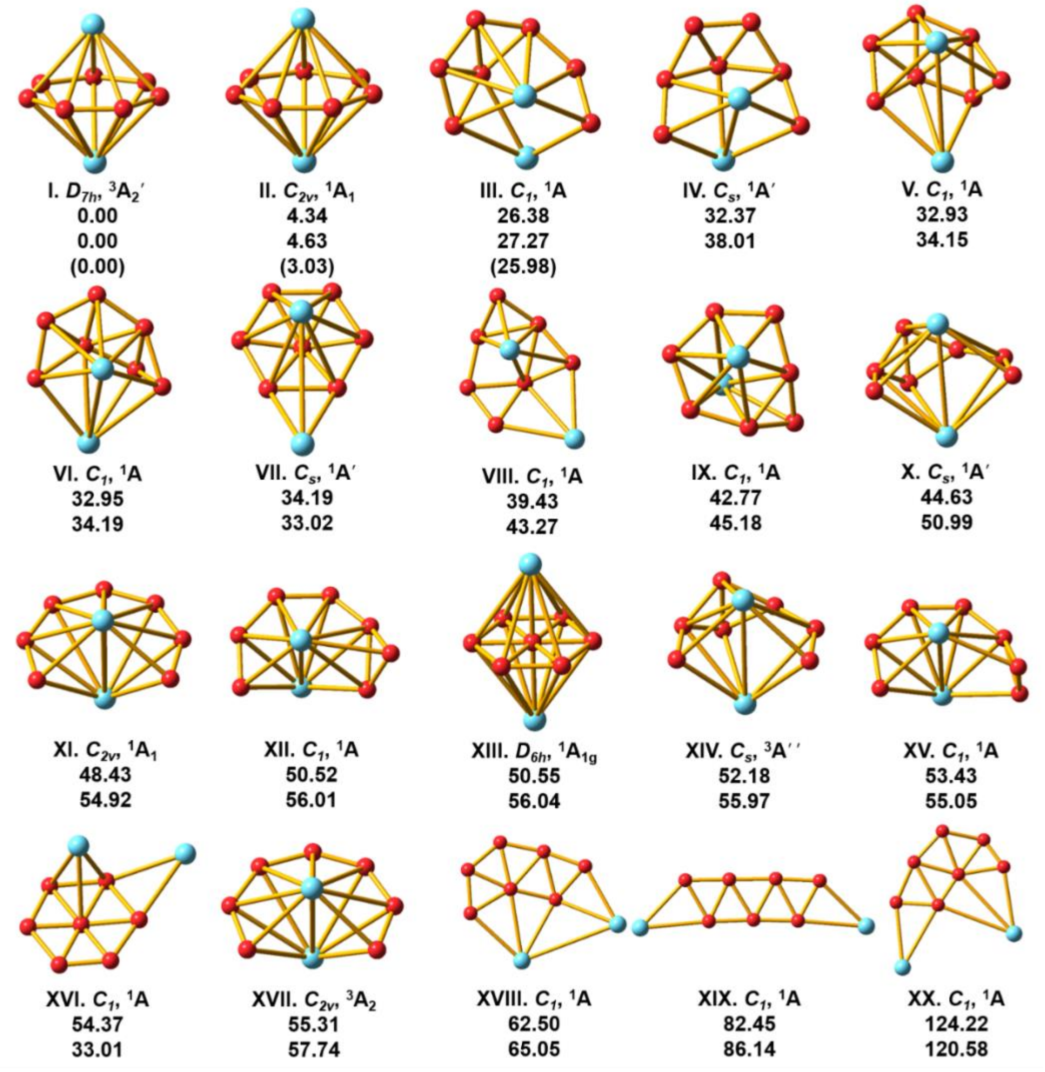

Fig. S1. Low-lying isomers of $La_2B_7^-$, their symmetries, and electronic states. The energies shown in kcal/mol are relative to the global minimum. The numbers in the first and second rows represent the energies obtained from the PBE and PBE0 levels, respectively. The CCSD(T) energies for the three lowest-lying isomers are given in the parentheses.

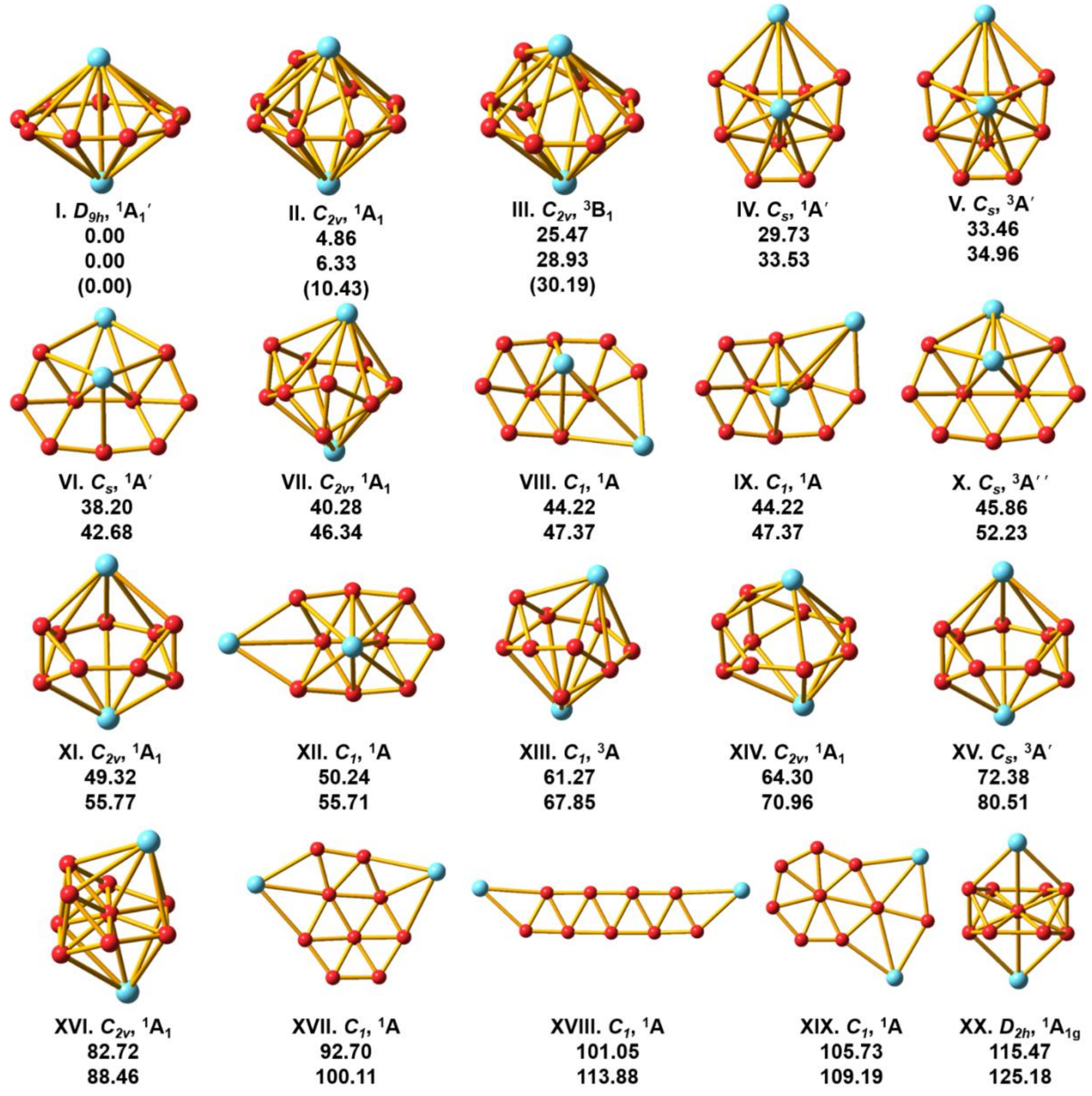

Fig. S2. Low-lying isomers of $La_2B_9^-$, their symmetries, and electronic states. The energies shown in kcal/mol are relative to the global minimum. The numbers in the first and second rows represent the energies obtained from the PBE and PBE0 levels, respectively. The CCSD(T) energies for the three lowest-lying isomers are given in the parentheses.

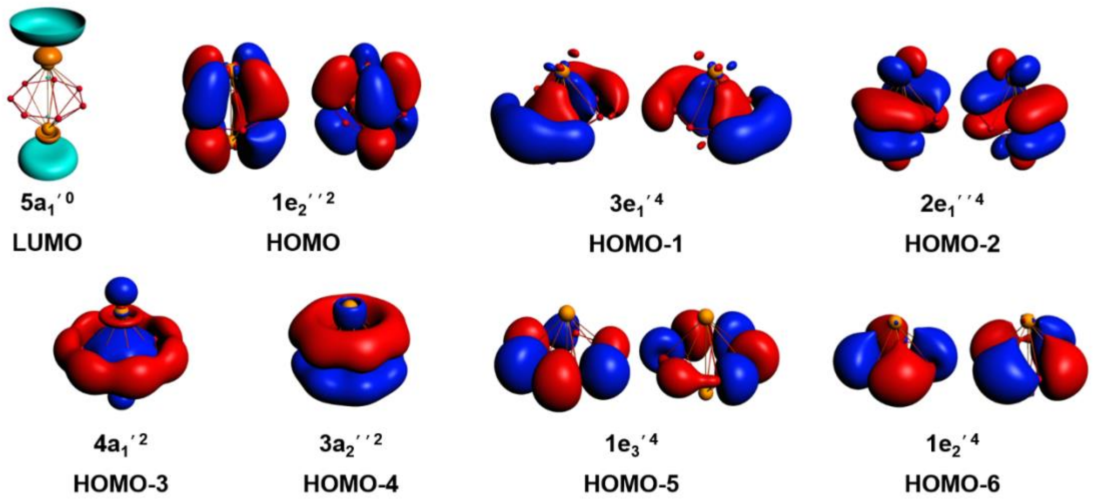

Fig. S3. Canonical molecular orbitals of $La_2B_7^-$ at the PBE0/TZP level (isovalue = 0.03 au).

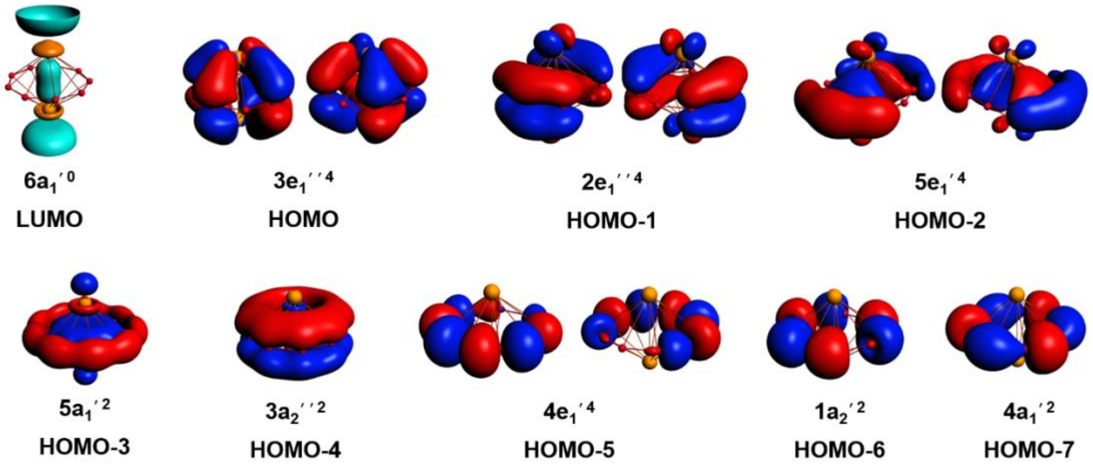

Fig. S4. Canonical molecular orbitals of $La_2B_9^-$ at the PBE0/TZP level (isovalue = 0.03 au).

Table S1. EDA-NOCV analyses of $La_2B_x^-$ (x = 7−9) at the PBE0/TZP level. Charge flows from red → blue.

	$La_2B_7^-$		$La_2B_8^-$		$La_2B_9^-$	
Fragment	$La_2(d\pi_u^4 d\delta_u^2)$ + $B_7^-(\pi_1^4\sigma_{r1}^0)$		$La_2(d\pi_u^3 d\delta_u^3)$ + $B_8^-(\pi_1^4\sigma_{r1}^1)$		$La_2(d\pi_u^2 d\delta_u^4)$ + $B_9^-(\pi_1^4\sigma_{r1}^2)$	
Spin	α	β	α	β	α	β
$\Delta E_{orb(1)}$	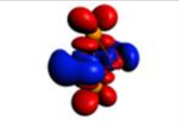	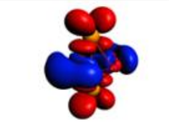	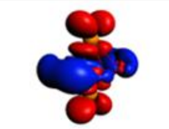	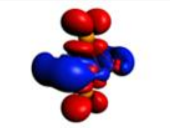	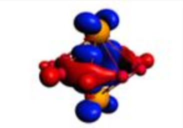	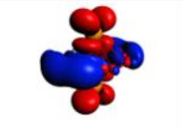
	-153.5 (18.5%)[a]	-156.8 (18.9%)	-152.1 (21.0%)	-153.2 (21.1%)	-12.5 (2.2%)	-121.0 (21.0%)
$\Delta E_{orb(1)'}$	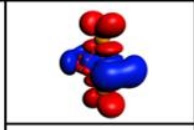	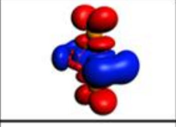	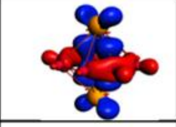	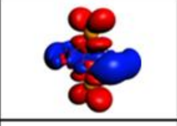	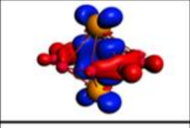	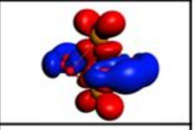
	-153.5 (18.5%)	-156.8 (18.9%)	-11.5 (1.6%)	-153.2 (21.1%)	-12.5 (2.2%)	-121.0 (21.0%)
$\Delta E_{orb(2)}$	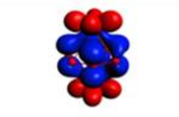	-	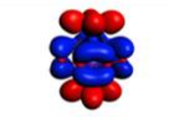	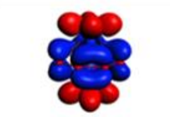	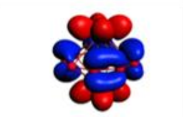	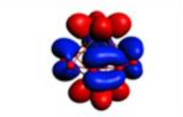
	-57.2 (6.9%)	-	-63.3 (8.7%)	-59.4 (8.2%)	-62.0 (10.8%)	-60.0 (10.4%)
$\Delta E_{orb(2)'}$	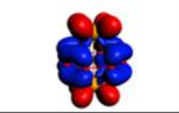	-	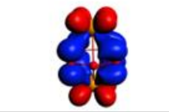	-	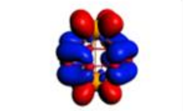	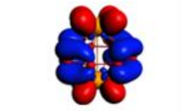
	-57.2 (6.9%)	-	-63.3 (8.7%)	-	-62.0 (10.8%)	-60.0 (10.4%)
$\Delta E_{orb(3)}$	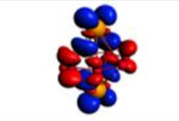	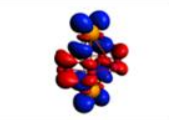	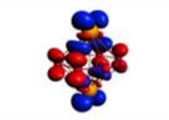	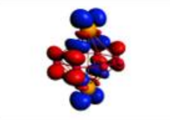	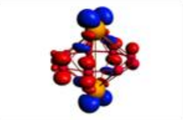	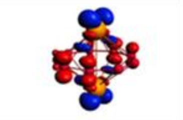
	-12.3 (1.5%)	-11.6 (1.4%)	-7.3 (1.0%)	-7.8 (1.1%)	-4.2 (0.7%)	-5.1 (0.9%)
$\Delta E_{orb(3)'}$	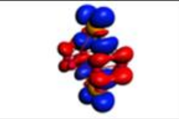	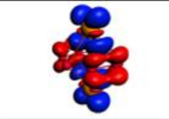	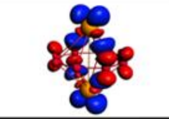	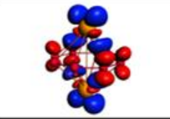	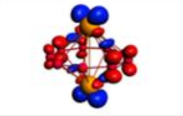	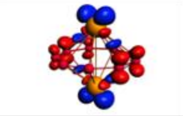
	-12.3 (1.5%)	-11.6 (1.4%)	-7.3 (1.0%)	-7.8 (1.1%)	-4.2 (0.7%)	-5.1 (0.9%)
$\Delta E_{orb(4)}$	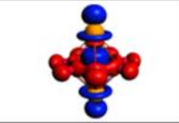	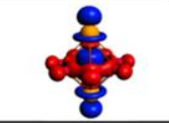	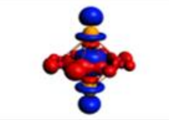	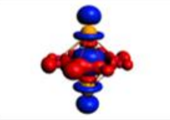	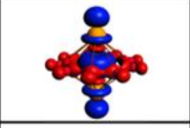	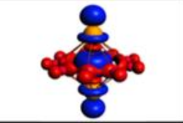
	-11.9 (1.5%)	-11.4 (1.4%)	-8.5 (1.2%)	-9.1 (1.3%)	-5.3 (0.9%)	-7.9 (1.4%)

[a] Values in parentheses correspond to the percentages of total orbital interactions .

Table S2. The calculated atomic charges on the La and B atoms in $La_2B_x^{-}$ (x = 7−9) with different partition schemes at the PBE0/TZP level.

	Mulliken		Hirshfeld		Voronoi		MDC-q	
	La	B	La	B	La	B	La	B
$La_2B_7^{-}$ [a]	0.366	-0.247	0.413	-0.261	0.493	-0.283	1.034	-0.438
$La_2B_8^{-}$ [b]	0.647	-0.287	0.475	-0.244	0.539	-0.260	1.029	-0.382
$La_2B_9^{-}$	0.722	-0.272	0.526	-0.228	0.578	-0.239	1.035	-0.341

[a] Spin densities on La and B of $La_2B_7^{-}$ are calculated to be 0.62 and 0.11, respectively.

[b] Spin densities on La and B of $La_2B_8^{-}$ are calculated to be 0.26 and 0.06, respectively.

Table S3. Bond distance, bond order index of La…La and binding energies (BE) between the two La atoms and the B_x^{-} rings in $La_2B_x^{-}$ (x = 7−9) at the PBE0/TZP level.

	La−La distance (Å)	La−La bond order			BE (kcal/mol)[a]
		Mayer	G-J	N-M	
$La_2B_7^{-}$	3.832	0.402	0.408	0.435	340.43
$La_2B_8^{-}$	3.646	0.428	0.376	0.401	367.84
$La_2B_9^{-}$	3.470	0.427	0.388	0.435	372.42

[a] Binding energies are calculated as follows:

$La_2B_7^{-}$: $La_2B_7^{-}$ (D_{7h}, $^3A_2'$) $\rightarrow$ 2La (^2D) + B_7^{-} (D_{7h}, $^1A_1'$);

$La_2B_8^{-}$: $La_2B_8^{-}$ (D_{4h}, $^2B_{2u}$) $\rightarrow$ 2La (^2D) + B_8^{-} (D_{4h}, ^2E_u);

$La_2B_9^{-}$: $La_2B_9^{-}$ (D_{9h}, $^1A_1'$) $\rightarrow$ 2La (^2D) + B_9^{-} (D_{9h}, $^3A_2'$).
